# The length of the embryo culture in assisted reproductive technology does not have a major impact on newborn DNA methylation

**DOI:** 10.1042/BSR20260093

**Published:** 2026-07-22

**Authors:** Jo Ciantar, Emma Raitoharju, Saara Marttila, Terho Lehtimäki, Eeva-Maria Pohjonen, Katja Ahinko, Noora Kaartinen

**Affiliations:** 1 Molecular Epidemiology (MOLE), Faculty of Medicine and Health Technology, Tampere University, Arvo Ylpön katu 34, Tampere 33520, Finland; 2 Finnish Cardiovascular Research Center Tampere, Faculty of Medicine and Health Technology, Tampere University, Arvo Ylpön katu 34, Tampere 33520, Finland; 3 Tays Research Services, Wellbeing Services County of Pirkanmaa, Tampere University Hospital, Tampere, Finland; 4 Gerontology Research Center, Tampere University, Tampere, Finland; 5 Department of Clinical Chemistry, Pirkanmaa Hospital District, Fimlab Laboratories, and Finnish Cardiovascular Research Center, Tampere, Faculty of Medicine and Health Technology, Tampere University, Arvo Ylpön katu 34, Tampere 33520, Finland; 6 Department of Obstetrics and Gynaecology, Tampere University Hospital, Wellbeing Services County of Pirkanmaa, Finland

**Keywords:** blastocyst, cleavage-stage embryo, epigenetics, EWAS, extended culture, methylation

## Abstract

Blastocyst culture is an essential part of IVF/ICSI treatments, however, it has been shown to increase the risk of preterm deliveries as well as large for gestational age infants. The effect of the extended culture on offspring phenotype is thought to be mediated by epigenetic mechanisms, since this developmental period coincides with extensive epigenetic remodelling. Here, we compare genome-wide DNA methylation of cord blood and umbilical cord artery samples collected from newborns resulting from cleavage-stage transfer (*n* = 25), blastocyst-stage transfer (*n* = 25), and spontaneous conception (*n* = 30). Epigenome-wide association studies, global methylation analyses, and epigenetic age comparison did not reveal statistically significant differences between the cleavage-stage and blastocyst-stage groups. However, we identified some loci with a >10% difference in median methylation level between the groups, which should be studied further with a larger sample size. The genome-wide DNA methylation of spontaneously conceived and cleavage-stage newborns were comparable while in the spontaneous to blastocyst-stage comparison, cg25263722 was differentially methylated (*P* = 5 × 10^−8^) in umbilical cord artery. Our study did not identify major effects of extended culture on DNA methylation, which is reassuring with respect to the future health of newborns conceived via assisted reproductive technologies.

## Introduction

The development of a preimplantation embryo culture from a zygote not only to the cleavage stage but also to the blastocyst stage has been a remarkable improvement in assisted reproductive technology (ART). *In vivo*, the embryo resides in the fallopian tube for 96–120 hours before entering the uterine cavity, reaching the cavity only 20–24 hours before implantation [1]. Therefore, blastocyst transfer can be perceived as more analogous to a natural conception compared to cleavage-stage transfer. Since only approximately 50% of embryos reach the blastocyst stage, blastocyst culture can also serve as a means of self-selection for viable embryos with good implantation potential, thus reducing the number of fruitless transfers [2]. Blastocyst transfer leads to a higher live birth rate than cleavage-stage transfer [3,4], however, it remains uncertain whether it benefits the cumulative clinical pregnancy rate.

In addition to developing methods to increase the chances of successful pregnancy, a major avenue of ART research is assessing the health of the offspring. Some concerns over the effect of extended culture on the health of the newborns have been raised; a significantly higher rate of preterm births (<37 weeks of pregnancy) and very preterm births (<32 weeks of pregnancy) has been detected after blastocyst transfer compared to cleavage-stage transfer [5–7]. Additionally, the neonates born from blastocyst transfer have an increased risk of being large for gestational age (LGA) [6,8–11], and some studies have reported a higher birth weight for babies born from blastocyst transfer [12–14].

The mechanisms by which extended embryo culture results in an increased risk of preterm birth or changes in birth weight are not clear, but epigenetic modifications, such as DNA methylation, have been proposed as molecular mediators [15–18]. DNA methylation is an essential epigenetic mechanism, regulating the tissue-specific gene expression by the attachment of a methyl group, typically to the cytosine of a cytosine–guanine pair (CpG). During the first days of embryonic development, the embryo undergoes genome-wide demethylation and subsequently *de novo* methylation [19]. During this period of extensive epigenetic remodelling, the embryos produced by means of ART are exposed to culture in an artificial environment, potentially causing epigenetic dysregulation. As most IVF media contain little to no methyl donors, the culture period has been hypothesised to affect the establishment and maintenance of DNA methylation patterns [20]. The blastocyst embryos stay in the culture conditions longer (5–6 days) than cleavage-stage embryos (2–3 days), which may intensify the possible effects of the artificial environment on the methylation pattern of the blastocysts. This perturbation in the process of epigenetic remodelling has been speculated to lead to transformations in the foetal growth trajectory and to changes in gestational length after blastocyst culture [10,21].

In the present study, we seek to identify differences in DNA methylation levels from cord blood and umbilical cord arteries of neonates born from blastocyst-stage and cleavage-stage embryo transfers. We performed an epigenome-wide association study (EWAS) and compared global methylation levels to detect any DNA methylation differences between the two groups. We also identified loci with a large difference in median methylation levels between the two groups. Additional analyses were also performed to detect epigenetic differences between the spontaneously conceived (SC) and cleavage- or blastocyst-transfer newborns.

## Methods

### Study participants

The cohort comprises 50 couples who had undergone IVF/ICSI treatment at the Tampere University Hospital Infertility Clinic and 30 couples with a spontaneous pregnancy recruited from the antenatal ward of Tampere University Hospital. Only singleton pregnancies with delivery after gestational week 36 + 6 were included in the final study material.

After delivery, cord blood samples were successfully collected for 76 (47 ART and 29 SC) newborns, while umbilical cord artery was collected from all 80 (50 ART and 30 SC) participants. Cord blood samples were collected in 10 ml EDTA tubes and 15-cm-long segments of the umbilical cords were stored in RNA-later solution. The cord blood was centrifuged and frozen and the umbilical arteries were separated from the other cord sample tissue and preserved in the freezer. Information regarding pregnancy-related diagnoses, pregnancy complications and the mode of delivery were collected from the patient records of the mother.

### DNA isolation and methylation measurements

DNA was isolated from cord blood samples using the PerkinElmer chemagic DNA Blood 4k kit and the chemagic 360 robot, following the manufacturer’s instructions. DNA extraction from the umbilical cord arteries was performed using the QIAGEN’s QIAamp DNA mini kit, following the protocol for DNA extraction from tissues. Genome-wide DNA methylation levels were determined using the Illumina Infinium MethylationEPIC BeadChip at Helmholtz Zentrum in Munich, Germany, following the manufacturer’s protocols.

### DNA methylation data processing

Raw DNA methylation values were processed separately for cord blood and umbilical cord arteries. The signal intensities were normalised using the *minfi* [22] and *limma* [23] R packages as described previously [24]. The sample call rate threshold was set at 90% with a detection *P-*value of ≤10^−16^ and resulted in the exclusion of one cord artery sample. Sex chromosomes, as well as sites overlapping with known SNPs and cross-reactive probes [25], were removed, leaving 659,336 and 619,390 probes for cord blood and cord arteries, respectively. Estimation of blood cell composition was performed on cord blood samples, using the cell composition estimation for cord blood function in *minfi*.

### Statistical analysis

Participant demographics were compared across the three groups using the Kruskall–Wallis rank sum test or the Fisher’s exact test for continuous and categorical variables, respectively. This analysis was performed in R using the *TableOne* package [26].

### Differential methylation analysis

An EWAS was performed between the newborns. When comparing the two ART groups, the linear regression model was adjusted with sex, gestational age, transfer type (fresh or frozen), blood cell composition (only for data derived from cord blood), and the first 30 principal components of the technical probes. The same covariates were used for the comparison between SC and each ART group, but transfer type was excluded and maternal and paternal ages were included since a statistical difference in parental ages was detected between the SC and ART groups (Table 1). The false discovery rate (FDR) was calculated and the threshold for significance was set to 0.05. The *OrderedList* R package [27] was used to determine whether there was a statistically significant overlap between the top EWAS results for cord blood and cord arteries. Differentially methylated region (DMR) and variably methylated region (VMR) analyses were performed using the *DMRcate* R package [28]. M methylation values were calculated, and the default settings were used, with the inclusion of the same covariates that were used in the EWAS.

### Measures of epigenetic age

Epigenetic ages were calculated for the Horvath [29], Lee [30], Bohlin [31], Knight [32], and EPIC [33] clocks using the *methylclock* Bioconductor R package [34]. The Horvath clock is a measure of chronological age, while the remaining clocks are measures of gestational age. Linear regression on the inverse normal transformed epigenetic age acceleration was used to determine whether there is a difference in ages between cleavage-stage-transfer, blastocyst-stage-transfer, or SC newborns. The model comparing the two ART groups was adjusted with sex, transfer type (fresh or frozen) and gestational age. The models comparing the SC and the ART groups were adjusted with the same covariates except for transfer type, and maternal and paternal ages were added. The difference in Horvath epigenetic ages between cord blood and cord arteries was determined using the paired samples Wilcoxin signed-rank test.

### Global methylation analysis

Linear regression was used to determine whether there is a difference between median DNA methylation levels of cleavage-, blastocyst-transfer, or SC newborns. Inverse normal transformation was applied to the median methylation values, and the regression models were adjusted with the same covariates as the EWAS described in the ‘Differential methylation analysis’ section.

### Methylation quantitative trait loci analysis

Since genetic data were not collected from our cohort, methylation quantitative trait loci (meQTLs) that associate with the methylation of loci of interest were identified by searching on the Genetics of DNA Methylation Consortium (GoDMC) database (available at: https://mqtldb.godmc.org.uk/). This database was created by a meta-analysis using a total of 32,851 participants across different cohorts to identify genetic variants that associate with genome-wide DNA methylation levels measured with the Illumina Epic 450K array [35].

### Differences in methylation levels between tissues

The difference in methylation levels between the cord blood and cord arteries of ART-conceived newborns was calculated using the Wilcoxin signed-rank test for dependent samples. The analysis was performed on participants from whom both tissues were collected (*n* = 46) and probes available for both tissues (617,832 probes).

## Results

### Demographics of the study population

The three newborn groups showed comparable demographics (Table 1), except in the parental age where both mothers (*P* = 0.03) and fathers (*P* = 0.005) of the SC group are younger than those of the two ART groups. When comparing the two ART groups, there is also a difference in the proportion of fresh and frozen-thawed embryos (*P*<0.001) with higher proportions of frozen-thawed embryos in the blastocyst-stage group. This discrepancy is due to the practice of transferring the fresh embryo at the cleavage stage, whereas the surplus embryos are often cultured to the blastocyst stage to enable the selection of viable embryos for freezing.

**Table 1:** Demographics of the Study Population

	Spontaneously conceived (*n* = 30)	Cleavage transfer (*n* = 25)	Blastocyst transfer (*n* = 25)	*P*-value
**Males**	12 (40)	12 (48)	14 (56)	0.496
**Gestational age (weeks)**	39.36 (38.61–39.93)	39.29 (39.00–40.14)	39.86 (38.86–40.43)	0.444
**Birthweight (g)**	3379 (3124–3572)	3470 (3140–3845)	3670 (3320–3896)	0.240
**Mother’s age (years)**	30 (26–34)	33 (30–36)	33 (31–36)	**0.030**
**Father’s age (years)**	31 (29–35)	35 (33–38)	35 (33–37)	**0.005**
**Mother’s BMI (kg/m^2^)**	25.25 (21.85–27.61)	23.74 (22.21–26.70)	24.46 (22.54–27.02)	0.757
**Father’s BMI (kg/m^2^)**	27.17 (24.76–30.43)	26.64 (23.51–28.06)	25.54 (23.67–27.46)	0.207
**Mother ever smoked**	9 (33)	9 (36)	12 (48)	0.519
**Father ever smoked**	13 (48)	12 (48)	11 (44)	0.945
**Delivery route**				
Vaginal	20 (67)	18 (72)	14 (56)	0.341
Vacuum extraction	4 (13)	2 (8)	1 (4)	
Cesarean section	6 (20)	5 (20)	10 (40)	
**Bleeding at delivery (g)**	450 (300–650)	500 (350–700)	450 (350–650)	0.627
**Gestational diabetes**				
Dietary	5 (17)	5 (20)	4 (16)	0.374
Medicated	5 (17)	0	4 (16)	
**Gestational hypertension**	4 (13)	2 (8)	2 (8)	0.744
**Pre-eclampsia**	1 (3)	2 (8)	0	0.326
**ART protocol**				
ICSI		12 (48)	12 (48)	1.000
IVF		13 (52)	13 (52)	
**Frozen embryo transfer**		2 (8)	18 (72)	**<0.001**
**Culture media**				
LG total		1 (4)	0	1.000
Origio SAGE 1-Step		24 (96)	25 (100)	
**Underlying infertility**				
Anovulation		2 (8)	4 (16)	0.330
Endometriosis		3 (12)	4 (16)	
Male factor only		6 (24)	10 (40)	
Multiple causes		5 (20)	1 (4)	
Tubal factor		1 (4)	0	
Unexplained		8 (32)	6 (24)	

The demographics of blastocyst-stage, cleavage-stage transfer, and SC participants included in the present study. Percentages are given in brackets while inter quartile ranges are given in square brackets. All the mothers who reported having ever smoked indicated that they quit smoking during the pregnancy except one participant who did not answer the question.

### No major changes in DNA methylation detected between newborns conceived by cleavage or blastocyst transfer

Genome-wide comparison of DNA methylation levels between cleavage- or blastocyst-transfer newborns revealed nominally significant (*P*<0.05) differential methylation at 31,180 and 25,540 CpG sites for cord blood and arteries, respectively (Supplementary Tables S1,S2). The top 10 hits for blood and arteries are shown in Table 2; however, none of the loci survived multiple testing correction (FDR <0.05). A comparison of the nominally significant CpGs between cord blood and cord arteries revealed an overlap of 1228 sites, 688 of which show the same direction of change (Supplementary Table S3). We did not identify a significant overlap between the results from cord blood and cord artery (weighted overlap score = 0; *P* = 0.032), suggesting that the results were not similar across the different tissues. Additionally, we did not identify any DMRs or VMRs between the groups. To the best of our knowledge, no open dataset containing the DNA methylation from ART-conceived newborns specifies the length of culture, therefore replication was not possible.

**Table 2:** Top 10 EWAS results for cord blood and cord artery

CpG	Chr	Locus	Gene name	Location	Beta	SE	*P*-value
**A**							
cg02360616	1	184943055	*FAM129A*	Gene body	0.020	0.001	2.7 × 10^−7^
cg03660365	17	79102708	*AATK*	Gene body	−0.015	0.001	1.3 × 10^−6^
cg03022094	6	11242059	*NEDD9*	Gene body	0.034	0.001	2.7 × 10^−6^
cg20827980	6	98165585	*/*	/	−0.037	0.002	4.7 × 10^−6^
cg17078912	14	37642356	*SLC25A21-AS1*	Gene body	0.034	0.002	5.4 × 10^−6^
cg10157184	1	112722391	*/*	/	−0.063	0.004	1.3 × 10^−5^
cg08323969	17	40770823	*/*	/	0.064	0.004	1.5 × 10^−5^
cg00317386	3	71594182	*FOXP1*	TSS1500; 5′UTR	−0.048	0.003	1.6 × 10^−5^
cg17055671	1	41234272	*NFYC*	Gene body	−0.027	0.002	1.6 × 10^−5^
cg21703586	4	122722613	*EXOSC9*	1st exon	0.009	0.001	1.8 × 10^−5^
**B**							
cg11231279	10	98561739	/	/	−0.071	0.009	3.0 × 10^−6^
cg18609343	7	99717180	*TAF6; CNPY4*	TSS1500; TSS200	0.018	0.002	3.9 × 10^−6^
cg08095502	8	99118864	*HRSP12*	Body	0.074	0.011	8.8 × 10^−6^
cg20818305	1	40222175	*PPIE*	3′UTR; Body	−0.026	0.004	1.9 × 10^−5^
cg15550144	1	212369463	*/*	/	−0.025	0.004	2.0 × 10^−5^
cg11471619	21	22770819	*NCAM2*	Body	0.058	0.009	2.1 × 10^−5^
cg03127174	8	15397731	*TUSC3*	5′UTR; 1st exon	−0.009	0.001	2.2 × 10^−5^
cg14363066	4	104104412	*CENPE*	Body	0.040	0.006	2.3 × 10^−5^
cg20319791	3	54180925	*CACNA2D3*	Body	0.059	0.010	2.8 × 10^−5^
cg16412341	2	227277087	/	/	0.047	0.008	3.8 × 10^−5^

An EWAS for cord blood (A) and cord artery (B) comparing cleavage-stage and blastocyst-stage transfer newborns did not reveal any major differences between the two groups. The ten loci with the smallest *P*-value from each tissue are shown. Genomic locations are listed in the GRCh37 build. TSS: transcription start site; UTR: untranslated region.

**Figure 1:**
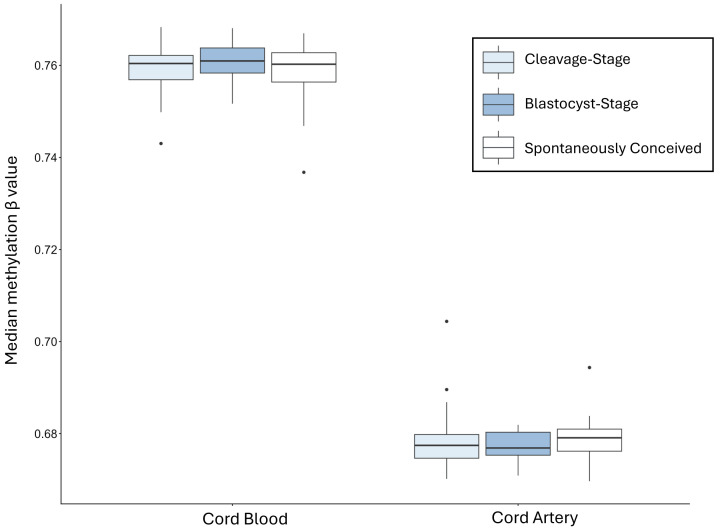
Differences in median methylation levels identified between cleavage- and blastocyst-transfer newborns Median DNA methylation levels of cleavage-stage, blastocyst-stage transfer and SC newborns are comparable for both cord blood and cord artery. The median methylation levels of cord blood (left) and cord artery (right) of the same participants are distinct (*P* = 2.84 × 10^−14^) showing differential methylation between the tissues.

Global DNA methylation levels were comparable for cleavage- and blastocyst-transfer (Figure 1) in both cord blood (*P* = 0.13) and cord arterial tissue (*P* = 0.31) and did not differ from the SC group. However, the median methylation levels between cord blood (methylation β ∼0.76) and cord arteries (methylation β ∼0.68) are markedly different (*P* = 2.84 × 10^−14^), demonstrating that there is a significant difference in methylation between different tissues for the same participants. Similarly, we found no difference in epigenetic ages between cleavage- and blastocyst-stage transfer with any of the epigenetic clocks (Supplementary Table S4) but found a significant difference (*P* = 2.84 × 10^−14^) in the calculated Horvath ages between the cord blood and cord artery samples of the same participants, highlighting the DNA methylation differences between these two tissues.

When investigating the median methylation levels at each CpG site between the two ART groups, our results show a >10% difference in median methylation levels between cleavage- and blastocyst-transfer newborns at 224 and 116 loci for cord blood and cord arterial tissue, respectively. Out of these sites, 44 were differentially methylated in both tissues and 43 had the same direction of change (Supplementary Table S5). Interestingly, 19 of the identified CpG sites are located in small clusters (<1000 bp), and all of the CpG sites in each cluster show the same direction of change (Table 3). The sites in question are six CpG sites in the gene body of *MUC4*, four CpG sites around 1500 bases upstream of the *ALLC* transcription start site, two CpG sites in the gene body of *HOOK2*, two CpG sites in an intergenic CpG island on chromosome 8, and three and two CpG sites in an intergenic region on chromosomes 14 and 6, respectively.

Since this analysis with not adjusted with covariates, we hypothesised that the results could have been influenced by differences in sex, gestational age, or whether the embryo was transferred fresh or frozen-thawed between the groups. However, only a few of the covariate *P*-values were nominally significant, and none reached genome-wide significance (*P*<5 × 10^−8^) for any of the 43 CpG sites (Supplementary Table S6).

We then investigated whether meQTLs, underlying genetic variants that affect DNA methylation, could play a causal role in the differential methylation at these sites by cross-checking the identified CpG sites with the GoDMC database [35] (available at: http://mqtldb.godmc.org.uk/). Out of the 43 sites, 23 had and 6 had not been previously identified to associate with a meQTL. For the remaining 14 sites, no data are available, since the GoDMC database is based on the older Illumina Infinium HumanMethylation450 BeadChip that includes only some 450,000 loci. The methylation at all of the 19 clustered CpG sites was found to associate with a genetic variant, save for two sites for which there are no data available (Table 3).

**Table 3:** Clusters of loci with a >10% difference in methylation levels

Gene	Location	Chr	Locus	CpG	Δβ cord blood (%)	Δβ cord artery (%)	meQTL *P*-value
*MUC4*	Gene body	3	195489306	cg05834845	−12.40	−15.20	<2.7 × 10^−322^
			195489708	cg13752114	−15.85	−17.29	<5.0 × 10^−324^
			195489782	cg18918831	−17.67	−11.19	<1.1 × 10^−322^
			195489789	cg18713687	−12.25	−11.96	<3.5 × 10^−323^
			195489909	cg07341007	−14.36	−10.35	<5.0 × 10^−324^
			195490033	cg01310397	−12.78	−10.07	<5.0 × 10^−324^
*ALLC*	TSS1500	2	3704530	cg19052272	15.68	14.29	<6.0 × 10^−315^
			3704589	cg10645314	17.00	11.48	<5.4 × 10^−240^
			3704751	cg00999904	15.74	10.55	<2.3 × 10^−318^
			3704773	cg25251562	22.30	12.02	<5.0 × 10^−324^
*HOOK2*	Gene Body	19	12876846	cg06417478	45.28	31.45	<5.0 × 10^−324^
			12877188	cg23899408	26.28	13.91	<6.0 × 10^−323^
/	CpG Island	8	599963	cg23958373	−10.75	−22.17	<1.2 × 10^−316^
			600039	cg07234876	−16.09	−26.02	<2.3 × 10^−320^
/	/	14	70690287	cg23618713	−14.33	−22.96	–
			70690296	cg23442650	−14.64	−14.70	–
			70690454	cg26146732	−12.31	−11.91	<2.9 × 10^−321^
/	/	6	29648604	cg03198009	20.40	11.42	<5.0 × 10^−324^
			29648628	cg15570656	12.67	11.25	<1.0 × 10^−323^

Six clusters (<1000 nucleotides) with ≥2 loci that have a >10% difference in median β methylation levels in the same direction between blastocyst-stage and cleavage-stage transfer newborns in both cord blood and cord artery were identified. The ∆β was calculated by subtracting the median value for blastocyst transfers from the median value for cleavage transfers. Data from the GoDMC database were used to find associating genetic variants and the lowest *P*-value is shown in the ‘meQTL P value’ column. No data were available for cg23618713 and 23442650. meQTL: methylation quantitative trait locus; TSS: transcription start site.

### DNA methylation of cleavage- and blastocyst-transfer newborns is mostly comparable to SC newborns

No statistically significant (FDR <0.05) loci were detected in the EWAS comparing SC and cleavage-stage transfers nor SC and blastocyst stage transfers in cord blood (Supplementary Table S7). In data from cord artery, a difference in cg25263722 methylation (*P* = 5 × 10^−8^) was identified between the blastocyst-transfer and SC newborns (Supplementary Table S8) however, the difference in group median methylation was only of 0.016. Global methylation analysis (Figure 1) showed no significant difference between SC and cleavage-transfer newborns (*P* = 0.52 in cord blood; *P* = 0.74 in cord artery) or SC and blastocyst-transfer newborn (*P* = 0.41 in cord blood; *P* = 0.96 in cord tissue). No difference in epigenetic ages were identified between the SC and either of the ART groups (Supplementary Table S9).

### Marked differences in DNA methylation patterns between cord blood and cord arteries

Since we identified such a significant difference in global DNA methylation levels between cord blood and cord arterial tissue of the newborns, we wanted to take a closer look at the tissue differences at the CpG level. Differential methylation analysis using the Wilcoxon signed-rank test for dependent samples revealed 458,044 significant CpG sites (FDR <0.05). Of these, 325,268 have a >2.5% difference in median methylation levels between the tissues (Supplementary Table S10). Approximately 60% of the differentially methylated CpG sites are hypermethylated in cord blood when compared to cord arterial tissue.

## Discussion

We conducted a thorough investigation to reveal potential perturbations in genome-wide DNA methylation caused by the extended exposure of embryos to the artificial milieu in *in vitro* culture in both cord blood and cord arterial tissue. Against our initial hypothesis, we did not detect any major effects of the extended culture on the methylation profile. Although numerous nominally differentially methylated sites were revealed in the EWAS, none of the loci survived multiple testing correction. We also identified some loci with large differences in median methylation levels between the cleavage-stage and blastocyst-stage transfer newborns, but these results may arise due to the confounding effect of an uneven distribution of genetic variants.

To the best of our knowledge, our study is the first to investigate the effect of extended culture on DNA methylation on a genome-wide scale. Previously, Ghosh et al. (2017) reported comparable global DNA methylation levels between cleavage- and blastocyst-stage transfers but differences between each ART group and SC [36]. Our analysis of global methylation levels between the three groups yielded no statistically significant results however, the previous study was performed using data from placenta while our analysis used cord blood and cord arterial tissue methylation levels.

Previously, DNA methylation differences have been detected in cord blood [37–40] and the placenta [36,41] between newborns born via ART and SC. There is also evidence of an effect of embryo freezing and thawing on methylation at three CpGs in cord blood [39], although another study in placenta found differences in global methylation levels only between SC and fresh transfers [36]. No methylation differences have been found between IVF and ICSI conception in cord blood [39,40] or the placenta [36]. Our study did not detect major methylation differences between the cleavage and blastocyst stage transfer or SC and cleavage stage transfer newborns neither in cord blood nor in cord arterial tissue. Only cg25263722 was found to be differentially methylated between SC-neonates and blastocyst transfer newborns in cord arterial tissue. The CpG site is located in the gene body of *EIF4G3*, which encodes a protein involved in 5′ cap-dependent mRNA translation [42]. Although this locus reached the threshold of statistical significance, the difference in median methylation level between the two groups is very small (<2%) that raises a question of its biological relevance. Moreover, our analysis contained data from 51 other CpGs in or near *EIF4G3*, none of which were found to be differentially methylated. Therefore, our results do not indicate a major effect of the blastocyst culture on DNA methylation when compared to SC, however, further research with larger samples sizes are needed to validate our findings.

DNA-methylation-based gestational age clocks have been developed to measure the effects of *in-utero* circumstances on the developing foetus [30–33]. Different factors that interfere with the DNA methylation of the developing foetus can also accelerate its epigenetic aging in comparison to chronological age. Both prenatal and birth characteristics are known to affect gestational age acceleration [43]. Previous research has not identified statistically significant differences in the gestational age acceleration of ART and SC newborns calculated from cord blood DNA methylation [33,37]. Similarly, our results indicate that an extended culture of the embryo does not affect the epigenetic ageing, further supporting our findings that an extended culture of the preimplantation embryo is not associated with major perturbations of the epigenome.

While extended culture has been associated with birthweight [9,12,13,44], and birthweight, in turn, has been associated with differences in DNA methylation profiles [45,46], we did not observe any major DNA methylation changes associated with extended culture. Other explanations for the altered neonatal outcomes besides epigenetic modifications have also been suggested. A higher male-to-female ratio after blastocyst transfer has been detected [47,48] and since preterm birth is more common in males, the increased incidence of preterm birth after blastocyst transfer could be partially explained by the skewed sex ratio [49,50]. The fresh blastocyst transfer embryos also derive from a slightly different group of patients than the fresh cleavage-stage transfers as it requires a rather good ovarian capacity with sufficient oocytes retrieved since only approximately 50% of the embryos reach the blastocyst stage [51]. The patient selection could partially explain the differences in the neonatal survival between babies from fresh blastocyst- and cleavage-stage transfers.

We included both cord blood and cord arteries in our analysis, as even though the DNA methylation of the child (cord blood) could be associated with health in later life, DNA methylation of the extra embryonic tissue could provide additional insight into the developmental stage and effects of pregnancy conditions [52]. We detected notable differences in global methylation levels between the tissues, as well as numerous differentially methylated loci. These differences are expected as different tissue and cell types require a different DNA methylation landscape in order to achieve cell-type specific gene-expression [53]. Previous studies have shown that pregnancy complications affect the methylation status with tissue-specificity. For instance, pre-eclampsia or gestational diabetes decrease the global methylation level in the placenta but not in the umbilical cord blood [54]. Given the tissue-specificity of DNA methylation patterns, results obtained from extraembryonic tissue most likely do not reflect the changes within the individual, suggesting tissues constituting the embryo proper would be preferable sample material in many research settings.

The strength of our study is the thorough and multifaceted exploration of the possible effects of blastocyst culture on the DNA methylation of newborns in both cord blood and cord arteries. Considering that the variety of methods used to identify DNA methylation differences between the two groups resulted in negative findings, it seems unlikely that the extended culture time has a major effect on DNA methylation.

The major drawback of our study is the small sample size. Due to the limited number of participants, more minor, yet biologically relevant, differences may remain undetected. It is also possible that longer culture time coupled with another condition could lead to DNA methylation changes or that more dramatic changes are only seen in a small minority of embryos. As other embryo culture conditions may potentially influence the embryo epigenome including culture media, oxygen tension and fertilisation method, these were kept constant between the two groups. A limitation of our study is the disproportion of the fresh and frozen transfers between groups—however, this was adjusted for in our statistical analyses.

Our results suggest that extended culture of an embryo does not promote major disturbances in the epigenetic reprogramming. The results are reassuring to the ART community, both to the future parents of children born from blastocyst culture and to the care providers.

## Supplementary Material

Supplementary Tables S1-S10

## Data Availability

Data used in this study comprise health-related participant data, and their use is therefore restricted under the regulations on professional secrecy (Act on the Openness of Government Activities, 612/1999) and on sensitive personal data (Personal Data Act, 523/1999, implementing the EU data protection directive 95/46/EC). Due to these legal restrictions, the data from this study cannot be stored in public repositories or otherwise made publicly available. The data are available upon reasonable request from the authors.

## Abbreviations

ART: assisted reproductive technology
CpG: Cytosine—phosphate—Guanine
DMR: differentially methylated region
EWAS: epigenome-wide association study
FDR: false discovery rate
GoDMC: Genetics of DNA Methylation Consortium
VMR: variably methylated region
meQTL: methylation quantitative trait loci
SC: spontaneous conception
IVF: invitro fertilisation
ICSI: intracytoplasmic sperm injection
